# Defining Levels of US Hospitals’ Pediatric Capabilities

**DOI:** 10.1001/jamanetworkopen.2024.22196

**Published:** 2024-07-15

**Authors:** Kenneth A. Michelson, Elizabeth R. Alpern, Katherine E. Remick, Rebecca E. Cash, Samaa Kemal, Courtney Benjamin Wolk, Carlos A. Camargo, Margaret E. Samuels-Kalow

**Affiliations:** 1 Division of Emergency Medicine, Ann & Robert Lurie Children’s Hospital, Northwestern University Feinberg School of Medicine, Chicago, Illinois; 2 Department of Pediatrics, Dell Medical School at the University of Texas at Austin; 3 Department of Emergency Medicine, Massachusetts General Hospital, Harvard Medical School, Boston; 4 Department of Psychiatry, Perelman School of Medicine, University of Pennsylvania, Philadelphia

## Abstract

**Importance:**

Classifying hospitals across a wide range of pediatric capabilities, including medical, surgical, and specialty services, would improve understanding of access and outcomes.

**Objective:**

To develop a classification system for hospitals’ pediatric capabilities.

**Design, Setting, and Participants:**

This cross-sectional study included data from 2019 on all acute care hospitals with emergency departments in 10 US states that treated at least 1 child per day. Statistical analysis was performed from September 2023 to February 2024.

**Exposure:**

Pediatric hospital capability level, defined using latent class analysis. The latent class model parameters were the presence or absence of 26 functional capabilities, which ranged from performing laceration repairs to performing organ transplants. A simplified approach to categorization was derived and externally validated by comparing each hospital’s latent class model classification with its simplified classification using data from 3 additional states.

**Main Outcomes and Measures:**

Health care utilization and structural characteristics, including inpatient beds, pediatric intensive care unit (PICU) beds, and referral rates (proportion of patients transferred among patients unable to be discharged).

**Results:**

Using data from 1061 hospitals (716 metropolitan [67.5%]) with a median of 2934 pediatric ED encounters per year (IQR, 1367-5996), the latent class model revealed 4 pediatric levels, with a median confidence of hospital assignment to level of 100% (IQR, 99%-100%). Of 26 functional capabilities, level 1 hospitals had a median of 24 capabilities (IQR, 21-25), level 2 hospitals had a median of 13 (IQR, 11-15), level 3 hospitals had a median of 8 (IQR, 6-9), and level 4 hospitals had a median of 3 (IQR, 2-3). Pediatric level 1 hospitals had a median of 66 inpatient beds (IQR, 42-86), level 2 hospitals had a median of 16 (IQR, 9-22), level 3 hospitals had a median of 0 (IQR, 0-6), and level 4 hospitals had a median of 0 (IQR, 0-0) (*P* < .001). Level 1 hospitals had a median of 19 PICU beds (IQR, 10-28), level 2 hospitals had a median of 0 (IQR, 0-5), level 3 hospitals had a median of 0 (IQR, 0-0), and level 4 hospitals had a median of 0 (IQR, 0-0) (*P* < .001). Level 1 hospitals had a median referral rate of 1% (IQR, 1%-3%), level 2 hospitals had a median of 25% (IQR, 9%-45%), level 3 hospitals had a median of 70% (IQR, 52%-84%), and level 4 hospitals had a median of 100% (IQR, 98%-100%) (*P* < .001).

**Conclusions and Relevance:**

In this cross-sectional study of hospitals from 10 US states, a system to classify hospitals’ pediatric capabilities in 4 levels was developed and was associated with structural and health care utilization characteristics. This system can be used to understand and track national pediatric acute care access and outcomes.

## Key Points

**Question:**

How can hospitals be sorted by level of pediatric capabilities?

**Findings:**

This cross-sectional study of hospitals from 10 US states found that hospitals could be sorted into 4 levels of pediatric capability based on the extent of services provided.

**Meaning:**

This study suggests that researchers and policymakers can compare outcomes and evaluate care delivery using this set of pediatric hospital capability levels.

## Teaser

This cross-sectional study of hospitals from 10 US states develops a classification system for hospitals’ pediatric capabilities, including medical, surgical, and specialty services.

## Introduction

Pediatric inpatient capabilities have been declining in US hospitals since at least 2008.^1,2^ This trend is due in part to inpatient unit closures, reductions in number of beds, and decreasing demand for inpatient pediatric care.^1,3^ As a result, there has been substantial regionalization of acute care pediatrics for patients across levels of medical complexity.^4,5^ Although these trends have not shown signs of slowing, national efforts to strengthen pediatric emergency readiness have successfully improved the readiness of emergency departments (EDs) to care for children over the long term, and readiness is associated with improved outcomes.^6,7,8,9,10^ This improvement in readiness was achieved in part through classification and improvement of EDs’ foundational resources and capabilities, or, in short, the development of a taxonomy of the resources needed for pediatric emergency care. No such classification system exists for a broader array of pediatric acute care capabilities outside the ED, complicating efforts to evaluate the effects of regionalization and access to pediatric acute care.

In the absence of an established taxonomy, researchers have used a variety of approaches to classify pediatric capabilities. These have included considering freestanding children’s hospital status,^11,12^ teaching status,^13^ volume of pediatric patients,^2,6^ structural features (such as presence of an inpatient unit or intensive care unit [ICU]),^1,14,15^ and propensity to transfer.^16^ However, none of these categories specifically identifies the extent of pediatric capabilities.^17^

The breadth of pediatric capabilities may best be defined by which services are actually performed in hospitals as opposed to the structures that exist. Similar to ED pediatric readiness, we hypothesize that pediatric capabilities are likely associated with pediatric outcomes.^10^ Classifying the functional pediatric capabilities of hospitals will allow for comparison of patient outcomes within more discrete categories of hospitals and facilitate comparison between regions. Our objective was to classify pediatric hospital capabilities based on functional phenotypes and to evaluate the association of phenotypes with hospitals’ structural characteristics, pediatric health care utilization, and referral practices.

## Methods

We first defined pediatric hospital capability phenotypes (ie, levels) using latent class analysis. We then performed a cross-sectional analysis evaluating the association of levels with structural and health care utilization characteristics. The Ann & Robert Lurie Children’s Hospital of Chicago institutional review board approved this study as exempt from review and with a waiver of informed consent because patients could not be identified. This study followed the Strengthening the Reporting of Observational Studies in Epidemiology (STROBE) reporting guideline.

### Setting and Participants

We included EDs in 2019 in Arkansas, Colorado, Florida, Georgia, Iowa, Maryland, Nebraska, North Carolina, New York, and Wisconsin, chosen for having high-quality health care utilization data. For encounter-level data, we included children younger than 15 years on the day of presentation, aligned with a previously suggested cutoff.^18,19^ We excluded EDs with an annual mean of fewer than 1 ED visit daily by children (as these hospitals have a negligible pediatric presence), having an annual median hospital length of stay of more than 14 days (to exclude long-stay and rehabilitation facilities), or for providing primarily psychiatric treatment (>50% of pediatric encounters with a primary mental or behavioral disorder diagnosis code based on the Healthcare Cost and Utilization Project [HCUP] Clinical Classifications Software code set). Each exclusion was selected because we have little expectation that the excluded hospitals will have capabilities to deliver significant pediatric acute care.

### Data Sources

For capability and health care utilization data, we used the HCUP State Emergency Department and Inpatient Databases. The HCUP databases include information for all ED and inpatient encounters in a state, including demographic characteristics, health care utilization data (eg, length of stay), diagnosis codes, and procedure codes. For structural data, we used the 2019 American Hospital Association (AHA) Survey. The HCUP provides linkages to the AHA Survey.

### Variables

For each hospital, we recorded the state, the proportion of patients with Medicaid, and urbanicity (urban, micropolitan, or rural, depending on the modal patient urbanicity from patient zip codes).^20^ Health care utilization measures were drawn from HCUP data and included ED, unplanned inpatient (hospitalizations originating in the ED), and ICU encounter counts; the proportion of all patients who were children (freestanding children’s hospitals generally have >70% pediatric encounters^21^); hospitalization rate; and referral rate (transfers divided by transfers plus hospitalizations, a measure of the propensity to transfer patients elsewhere who cannot be discharged^4^).

Structural characteristics were obtained from AHA data and included pediatric inpatient and ICU bed counts and freestanding children’s hospital status (defined by the AHA as restricting admissions only to children). Structural characteristics were measurable only for a subset of hospitals because AHA database linkage was not available for all HCUP hospitals. For analysis of structural features, we excluded those without available linkage and hospitals in which more than 1 HCUP hospital linked to 1 AHA hospital (in which the beds could not accurately be assigned to individual facilities).^22^

### Pediatric Capabilities

In our conceptual model of pediatric capabilities, we posited that acute care capabilities included procedures (eg, computed tomography scan or radius fracture reduction), levels of nursing care (eg, ICU), surgical procedures (eg, appendectomy), and subspecialties (eg, cystic fibrosis hospitalization as representing a pulmonary hospital capability). Neonatal capabilities are distinct from pediatric acute care, so we did not attempt to classify this separate set of services.^23^ The set of capabilities that we evaluated was based on author consensus. The authorship team includes health services researchers (all authors) and clinicians trained in general pediatrics (K.A.M., E.R.A., K.E.R., and S.K.), pediatric emergency medicine (K.A.M., E.R.A., K.E.R., and M.E.S.-K.), general emergency medicine (M.E.S.-K.), internal medicine (C.A.C.), and a current pediatric emergency medicine trainee (S.K.). We built a list of 26 capabilities ranging from common to subspecialized (Table 1). We mapped capabilities to diagnosis, procedure, and revenue code sets using 2019 codebooks (eTable in Supplement 1). A hospital was defined as having a given capability if it was used at least 3 times in a year by children. The threshold was chosen based on an analysis of the distribution of capability counts across the services.

**Table 1.** Characteristics of 1061 Acute Care Hospitals in 10 US States

Hospital characteristic	No. (%) (N = 1061)
State	
Arkansas	68 (6.4)
Colorado	67 (6.3)
Florida	206 (19.4)
Georgia	125 (11.8)
Iowa	100 (9.4)
Maryland	49 (4.6)
North Carolina	112 (10.6)
Nebraska	35 (3.3)
New York	187 (17.6)
Wisconsin	112 (10.6)
Urbanicity	
Metropolitan	716 (67.5)
Micropolitan	159 (15.0)
Rural	186 (17.5)
Health care utilization and structural characteristics^a^	
Proportion of children with Medicaid, median (IQR), %	67 (56-75)
Proportion of pediatric patients, median (IQR), %	13 (10-16)
Pediatric ED encounters per year, median (IQR)	2934 (1367-5996)
Hospitalization rate, median (IQR), %	0 (0-1)
Referral rate, median (IQR), %	94 (59-100)
Pediatric inpatient beds, median (IQR)	0 (0-8)
Pediatric intensive care beds, median (IQR)	0 (0-0)
Capabilities (performed ≥3/y)	
Appendectomy	455 (42.9)
Asthma hospitalization	405 (38.2)
Cerebral ventricular shunt	45 (4.2)
Chemotherapy hospitalization	60 (5.7)
Closed reduction of radius	651 (61.4)
Complex hospitalization	359 (33.8)
Congenital heart disease hospitalization	47 (4.4)
Computed tomography scan	1056 (99.5)
Cystic fibrosis hospitalization	48 (4.5)
Diabetic ketoacidosis hospitalization	124 (11.7)
Dialysis	37 (3.5)
ECMO	34 (3.2)
Endoscopy	125 (11.8)
Fontan surgery	21 (2.0)
Gastrostomy	155 (14.6)
ICU hospitalization	201 (18.9)
Inflammatory bowel disease hospitalization	75 (7.1)
Lumbar puncture	375 (35.3)
Mechanical ventilation	212 (20.0)
Organ transplant	25 (2.4)
Pneumonia hospitalization	387 (36.5)
Sickle cell anemia hospitalization	139 (13.1)
Simple laceration repair	984 (92.7)
Unplanned hospitalization	538 (50.7)
Urea cycle defect hospitalization	23 (2.2)
Ventricular septal defect surgery	23 (2.2)

Abbreviations: ECMO, extracorporeal membrane oxygenation; ED, emergency department; ICU, intensive care unit.

^a^ Structural data were available for 675 hospitals.

### Statistical Analysis

Statistical analysis was performed from September 2023 to February 2024. Using the HCUP data, we grouped hospitals by common sets of capabilities using latent class analysis.^24^ This method was chosen to avoid presupposing which capabilities would be grouped together; instead, hospitals were clustered using inherent groupings. Our modeling approach adhered to best practices for latent class analysis^25^: (1) we tested latent class model sets between 1 and 10 classes (“pediatric levels”) and selected the number of levels at which the bayesian information criterion stopped decreasing by less than 1% (the “elbow”)^25^; (2) for each model set, 100 models were tested with random starting parameters with 10 000 iterations each; (3) hospitals were assigned to a level based on the class with the maximum probability; (4) we reported the distribution of capabilities within each level so readers could judge their face validity; and (5) we prespecified which structural outcomes we would evaluate by level. Levels were ordered in descending order of counts of capabilities; the highest possible level would be a class where member hospitals had every capability.

We summarized the confidence of hospital assignments to level using the median and IQR modal estimated probability for level and the proportion of hospitals under a prespecified high-confidence probability threshold of 75%. In that group of less-certain hospitals, we determined the frequency of the next-best assignment being an adjacent level.

We determined the number of capabilities by level and the proportion of hospitals with each capability. Health care utilization and structural characteristics were also summarized by level.^25^

All *P* values were from 2-sided tests, and results were deemed statistically significant at *P* < .05. The analysis was conducted using R, version 4.3.0 (R Project for Statistical Computing), with the following main packages: poLCA, version 1.6.0.1 for latent class analysis; ggplot2, version 3.4.2 for graphical output; and data.table, version 1.15.0 for data management.

#### Development and Validation of Simplified Hospital Level Definitions

To simplify the hospital classifications, we created more easily applied level definitions. To do so, we assessed the range of the number of capabilities in the hospitals in each level and the services that were nearly universal in the level and qualitatively evaluated borderline cases. From this, we developed the list of capabilities that would define each simplified level. We determined the number of recategorized hospitals. Then, we externally validated the simplified definitions using 2018 HCUP data from 3 states: Arizona, New Jersey, and Nevada—distinct states during a distinct time period.^26^ We first used the comprehensive latent class model that we derived in the main analysis to assign a “true” level to each hospital. We then determined the proportion of hospitals that would be misclassified under the simplified definitions. We prespecified that a less than 10% misclassification rate was acceptable to adopt the simplified definitions.

An additional exploratory hypothesis was that a 4-level structural classification scheme could approximate the functional levels that emerged from latent classes. The use case of such a scheme would be for use in data sources containing only structural data but not functional data. The 4 prespecified levels were a freestanding children’s hospital, hospitals with at least 1 pediatric intensive care unit (PICU) bed, hospitals with at least 1 pediatric inpatient bed, and hospitals in none of those categories. We mapped each pediatric level to a structural category based on the modal pediatric level by structural type. We reported the proportion of hospitals correctly classified using a proportion with binomial 95% CIs.

#### Sensitivity Analyses

We performed 2 sensitivity analyses. First, we repeated the main analysis using a lower age cutoff, this time including only children younger than 12 years.^19^ The goal was to define the population of children more specifically. We determined the proportion of hospitals with unchanged functional classification. Second, we repeated the main analysis substituting a count threshold of 10 or greater to be labeled as having a given capability (eg, ≥10 unplanned hospitalizations would count as being capable instead of ≥3 unplanned hospitalizations).

## Results

Among 1331 hospitals (all hospitals in the included states in the HCUP database), we excluded 176 (13.2%) for having an annual mean of fewer than 1 ED visit daily by children, 9 (0.7%) for a median hospital length of stay more than 14 days, and 85 (6.4%) for being primarily psychiatric. This resulted in a final sample of 1061 hospitals (79.7% of the original sample), of which 716 (67.5%) were in metropolitan areas, with a median of 2934 pediatric encounters per year (IQR, 1367-5996), median of 0 pediatric inpatient beds (IQR, 0-8), and median referral of 94% (IQR, 59%-100%) of pediatric ED encounters not resulting in discharge (Table 1).

The latent class analysis with the best fit yielded 4 levels. The median confidence of level assignment was 100% (IQR, 99%-100%), but 55 hospitals (5.2%) had an assignment confidence under 75%. A total of 48 hospitals (4.5%) were classified as level 1, 116 (10.9%) as level 2, 308 (29.0%) as level 3, and 589 (55.5%) as level 4 (eFigure 2 in Supplement 1). Of the 55 moderate-confidence hospitals, the next most likely assignment was the next less-capable level in 20 (36.4%) and the next more-capable level in 35 (63.6%). There was 100% confidence between the 2 most likely levels.

The median number of pediatric capabilities was 24 (IQR, 21-25) for level 1 hospitals, 13 (IQR, 11-15) for level 2 hospitals, for level 3 hospitals was 8 (IQR, 6-9), and 3 (IQR, 2-3) for level 4 hospitals (Figure). Computed tomography scan and simple laceration repair were nearly universal across hospitals, including level 4. Most level 3 hospitals additionally were capable of unplanned pediatric hospitalizations, closed radius reduction, asthma hospitalization, pneumonia hospitalization, complex hospitalization, and appendectomy. Most level 2 hospitals additionally hospitalized children with sickle cell anemia and diabetic ketoacidosis and performed intensive care, lumbar puncture, endoscopy, gastrostomy, and mechanical ventilation. Level 1 hospitals added the remaining capabilities ranging from inflammatory bowel disease hospitalization (100%) to Fontan surgery (43.8% [21 of 48]).

**Figure.**
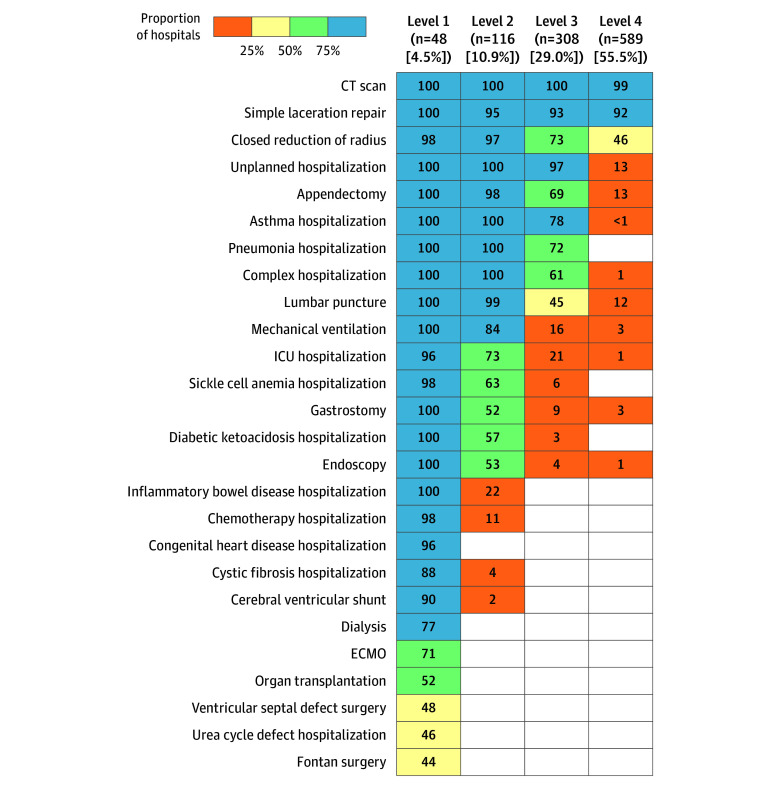
Hospital Functional Capabilities by Level Percentages of hospitals by functional level that had each capability, as defined by performing a procedure 3 or more times in 2019 (blue, ≥75%; green, 50% to <75%; yellow, 25% to <50%; orange, >0% to <25%; white, 0%). Functional levels were determined using latent class analysis. CT indicates computed tomography; ECMO, extracorporeal membrane oxygenation; and ICU, intensive care unit.

Structural data were available for 675 (63.6%) of the 1061 hospitals. Health care utilization and structural characteristics by level are shown in Table 2 and graphically in eFigure 1 in Supplement 1. Hospitals had higher volumes and capacities with decreasing functional hospital level. Pediatric level 1 hospitals had a median of 66 inpatient beds (IQR, 42-86), level 2 hospitals had a median of 16 (IQR, 9-22), level 3 hospitals had a median of 0 (IQR, 0-6), and level 4 hospitals had a median of 0 (IQR, 0-0) (*P* < .001). Level 1 hospitals had a median of 19 PICU beds (IQR, 10-28), level 2 hospitals had a median of 0 (IQR, 0-5), level 3 hospitals had a median of 0 (IQR, 0-0), and level 4 hospitals had a median of 0 (IQR, 0-0) (*P* < .001). Level 1 hospitals had a median referral rate of 1% (IQR, 1%-3%), level 2 hospitals had a median of 25% (IQR, 9%-45%), level 3 hospitals had a median of 70% (IQR, 52%-84%), and level 4 hospitals had a median of 100% (IQR, 98%-100%) (*P* < .001).

**Table 2.** Structural and Health Care Utilization Measures by Pediatric Capability Level

Measure	Median (IQR) value			
	Level 1	Level 2	Level 3	Level 4
ED encounters (thousands)	24 (15-35)	10 (7-17)	4 (2-6)	2 (1-4)
Hospitalization rate, %	8 (6-11)	3 (2-5)	1 (0-2)	0 (0-0)
ICU hospitalizations	960 (452-1361)	18 (2-148)	0 (0-2)	0 (0-0)
Pediatric inpatient beds	66 (42-86)	16 (9-22)	0 (0-6)	0 (0-0)
Pediatric ICU beds	19 (10-28)	0 (0-5)	0 (0-0)	0 (0-0)
Pediatric patients, %	23 (16-34)	14 (12-17)	13 (10-15)	13 (9-16)
Referral rate, %	1 (1-3)	25 (9-45)	70 (52-84)	100 (98-100)
Unplanned hospitalizations	1655 (1180-3222)	290 (146-694)	24 (10-66)	0 (0-1)

Abbreviations: ED, emergency department; ICU, intensive care unit.

### Development and Validation of Simplified Definitions of Levels

The simplified functional definitions of hospital levels are shown in the Box. In the main dataset from which the latent class model and the simplified definitions were derived, 1000 of 1061 hospitals (94.3%) were correctly classified using the criterion standard of the latent class assignments. In the external validation dataset from 3 states, there were 189 hospitals. The latent class model assigned 15 of 189 (7.9%) as level 1, 19 of 189 (10.1%) as level 2, 44 of 189 (23.3%) as level 3, and 111 of 189 (58.7%) as level 4. The simplified definitions correctly classified 184 of the 189 hospitals (97.4%; 95% CI, 93.9%-99.1%), indicating that the simplified definitions were an acceptable approximation of the comprehensive latent class model.

Box. **Simplified Definitions of 4 Pediatric Hospital Levels Based on Functional Capabilities**

**Level 1**

Capable of at least 7 of:

- Cerebral ventricular shunt
- Chemotherapy hospitalization
- Congenital heart disease hospitalization
- Cystic fibrosis hospitalization
- Diabetic ketoacidosis hospitalization
- Dialysis
- Endoscopy
- Extracorporeal membrane oxygenation
- Fontan surgery
- Gastrostomy
- Inflammatory bowel disease hospitalization
- Intensive care unit hospitalization
- Organ transplant
- Sickle cell anemia hospitalization
- Urea cycle defect hospitalization
- Ventricular septal defect surgery

**Level 2**

Does not meet level 1 criteria above and capable of at least 7 of:

- Total of capabilities from level 1 list PLUS:
- Appendectomy
- Closed reduction of radius
- Complex hospitalization
- Lumbar puncture
- Mechanical ventilation
- Pneumonia hospitalization

**Level 3**

Does not meet criteria for levels 1 and 2 above AND capable of at least 4 of:

- Total of capabilities from level 1 and 2 lists PLUS:
- Asthma hospitalization
- Unplanned hospitalization

**Level 4**

Does not meet criteria for levels 1 to 3 above

With the use of the exploratory prespecified 4-level structural classification system, 6 of 6 freestanding children’s hospitals (100.0%; 95% CI, 54.1%-100.0%) corresponded to level 1, 33 of 47 hospitals with PICU beds (70.2%; 95% CI, 55.1%-82.7%) corresponded to level 2, 92 of 169 hospitals with pediatric inpatient beds (54.4%; 95% CI 46.6%-62.1%) corresponded to level 3, and 342 of 453 hospitals with no pediatric beds (75.5%; 95% CI, 71.3%-79.4%) corresponded to level 4. Of the 675 hospitals with structural data, 473 (70.1%; 95% CI, 66.5%-73.5%) were correctly classified compared with the criterion standard latent class model.

### Sensitivity Analyses

In the sensitivity analysis recreating the latent class model using capability data with a lower age cutoff of younger than 12 years, there again were 4 levels. The classification was unchanged for 988 of 1061 hospitals (93.1%). In the sensitivity analysis with a higher threshold defining capability, 858 of 1061 hospitals (80.9%) had an unchanged classification. In this analysis, 140 of 203 reclassifications (69.0%) involved a level 3 hospital being reclassified as a level 4 hospital. Overall, the sensitivity analyses generally downgraded hospitals because of stricter entry criteria but preserved a 4-level classification scheme (eFigure 2 in Supplement 1).

## Discussion

In 1061 hospitals across 10 states, the functional characteristics of hospitals clustered into 4 levels of pediatric capability, which in turn were associated with structural characteristics such as inpatient unit size. We externally validated a simplified system of categorizing hospitals, creating a practical method for applying our findings. Thus, this study establishes a practically useful strategy for categorizing the spectrum of pediatric services performed by hospitals.

More than half of the hospitals (55.5%) were in level 4, the lowest-capability category, defined as performing only basic acute care services for children (such as laceration repair or computed tomography scan). Only 4.5% of hospitals were categorized as level 1, defined by having a broad spectrum of specialized services, such as hospitalization of children with cystic fibrosis or performing pediatric heart surgery. Although most children do not need such specialized services, when they are needed, they are often far from home.^27^ In addition, during pediatric disasters or volume surges, the few high-capability centers that exist may have limited flexibility to treat critically ill or injured patients, as was seen regionally during the fall 2022 viral respiratory epidemic.^28,29,30^

Pediatric capability levels are analogous to trauma center levels. Although pediatric capability levels have not yet been associated with outcomes, pediatric trauma center status is associated with improved trauma mortality and functional outcomes.^31,32,33,34^ Designation or verification as a pediatric trauma center is rigorous and has both functional requirements (eg, minimum volumes) and structural requirements (eg, presence of certain specialty resources).^35^ The levels that we propose are focused on medical, not trauma, capabilities because trauma designation processes are already robust.

This classification of capabilities has important implications. Similar to the trauma and ED contexts, conceptualization of pediatric capability levels would allow for comparison of outcomes by capability and, if shown to be associated with outcomes, could lead to a process for pediatric capability designation and stronger regional cooperation within the pediatric acute care system.^36,37^ Researchers can use this system to compare outcomes within and between hospital types, assess regionalization, evaluate the geography of pediatric acute care access (eg, proximity to high-capability hospitals), recruit patients by hospital context, assess regional pediatric network adequacy, or answer other clinical or health services questions. If this system is shown to be associated with relevant outcomes, policymakers could use this system to evaluate pediatric capacity, assess surge and disaster plans, and, potentially, designate status as in the trauma and readiness contexts. For the latter to occur, external entities would need to verify this system. We believe the simplified system or the structural approximation (freestanding children’s hospital, PICU presence, inpatient unit presence, or none of the above) are reasonable approaches to applying this system, and the choice would depend on the data source.

As with all taxonomies, this classification system depends on certain design decisions. First, the threshold of performing 3 procedures to qualify as capable is a low bar. However, even at this low threshold, for many procedures, the most common count was zero. We chose the threshold based on a visual inspection of the distributions of procedure counts. The sensitivity analysis increasing the threshold to 10 resulted in fewer level 1 hospitals and more level 4 hospitals but preserved the 4-level classification scheme, suggesting the robustness of a 4-level scheme. Second, the choice of counting only patients younger than 15 years was made with the goal of differentiating procedures that are sometimes performed among young adults because of similar physiology. Adult anatomy and physiology are not a binary, so no one cutoff would perfectly balance sensitivity and specificity for pediatric-specific capabilities. We tested the importance of this age threshold using an age cutoff of 12 years, which yielded a similar classification scheme as in the main analysis.

### Limitations

This study had several limitations. First, procedure coding is imperfect, particularly among patients discharged from the ED, leading some high-capability hospitals to seemingly lack basic services (such as closed radius reduction). This was uncommon, however, and we believe it was mitigated using our low threshold for determining that a capability was present. Second, we did not evaluate neonatal capabilities, which are distinct from hospital capabilities for older children. We plan to extend this effort to evaluate neonatal capabilities as a distinct domain of services in the future. Third, although we identified inherent groupings of functional characteristics, the importance of this classification can be established only by evaluating its association with outcomes. Comparison of functional and structural classification approaches should be undertaken to understand which approaches are most important. Fourth, in some cases, hospital campuses are grouped in the data source as 1 hospital system, making it difficult to discern the capabilities of single-hospital facilities. This scenario would be unlikely to affect the latent class analysis because advanced capabilities would tend to occur at 1 campus that would be represented as a whole hospital system. However, it could lead to underestimates in the proportion of low-capability hospitals.

## Conclusions

This cross-sectional study found that hospitals’ functional characteristics clustered into 4 levels of pediatric capability, which can be approximated by counting the number and types of capabilities at a hospital. These pediatric capability levels define the breadth of services offered to children at each hospital. This system will support research comparing pediatric acute care outcomes and evaluating care delivery.

## Notes

Supplement 1. **eTable.** Pediatric Capability Definitions **eFigure 1.** Utilization of Pediatric Resources by Hospitals in Each Pediatric Level **eFigure 2.** Classification of Hospitals Into Functional Levels in the Main Analysis and in Two Sensitivity Analyses

Supplement 2.
Data Sharing Statement
