# Supplementary material for: Defining Levels of US Hospitals’ Pediatric Capabilities
Source: JAMA Netw Open. 2024 Jul 15;7(7):e2422196. doi: 10.1001/jamanetworkopen.2024.22196 (PMC11250363; doi:10.1001/jamanetworkopen.2024.22196)
Supplement: Supplement 1. — eTable. Pediatric Capability Definitions eFigure 1. Utilization of Pediatric Resources by Hospitals in Each Pediatric Level eFigure 2. Classification of Hospitals Into Functional Levels in the Main Analysis and in Two Sensitivity Analyses [file jamanetwopen-e2422196-s001.pdf]

Supplementary Online Content

Michelson KA, Alpern ER, Remick KE, et al. Defining levels of US hospitals' pediatric capabilities. *JAMA Netw Open.* 2024;7(7):e2422196. doi:10.1001/jamanetworkopen.2024.22196

eTable. Pediatric Capability Definitions
eFigure 1. Utilization of Pediatric Resources by Hospitals in Each Pediatric Level
eFigure 2. Classification of Hospitals Into Functional Levels in the Main Analysis and in Two Sensitivity Analyses

This supplementary material has been provided by the authors to give readers additional information about their work.

© 2024 Michelson KA et al. *JAMA Network Open.*

eTable. Pediatric Capability Definitions
Codes ending in * indicate any code beginning with the given stem. In general, CPT codes only appear in patients discharged from the ED, so CPT codes were not given for surgeries routinely done for inpatients.

Capability	System	Codes
Appendectomy	ICD-10-PCS	0DBJ0ZX, 0DBJ0ZZ, 0DBJ3ZX, 0DBJ3ZZ, 0DBJ4ZX, 0DBJ4ZZ, 0DBJ7ZX, 0DBJ7ZZ, 0DBJ8ZX, 0DBJ8ZZ, 0DTJ0ZZ, 0DTJ4ZZ, 0DTJ7ZZ, 0DTJ8ZZ
	CPT	44950, 44960, 44970
Asthma hospitalization	CCS refined	J45*
Cystic fibrosis hospitalization	ICD-10-CM	E84*
Congenital heart disease hospitalization	ICD-10-CM	Q213*, Q220*, Q234*
Chemotherapy hospitalization	ICD-10-CM	Z511*
CT scan	CCS	177, 178, 179, 180
	CPT	70450, 70460, 70470, 70480, 70481, 70482, 70486, 70487, 70488, 70490, 70491, 70492, 70496, 70498, 71250, 71260, 71270, 71271, 71275, 72125, 72126, 72127, 72128, 72129, 72130, 72131, 72132, 72133, 72191, 72192, 72193, 72194, 73200, 73201, 73202, 73206, 73700, 73701, 73702, 73706, 74150, 74160, 74170, 74174, 74175, 74176, 74177, 74178, 7426*
	Revenue code	350, 351, 352, 359
Dialysis	ICD-10-PCS	3E1M39Z, 5A1D00Z, 5A1D60Z, 5A1D70Z, 5A1D80Z, 5A1D90Z
Diabetic ketoacidosis hospitalization	ICD-10-CM	E101, E1010, E1011, E111, E1110, E1111, E131, E1310, E1311
ECMO	ICD-10-PCS	5A1522F, 5A1522G, 5A1522H
	CPT	33946, 33947, 33948, 33949, 33951, 33952, 33953, 33954, 33955, 33956, 33957, 33958, 33959, 33962, 33963, 33964, 33966
Endoscopy	ICD-10-PCS	0DB18ZX, 0DB18ZZ, 0DB28ZX, 0DB28ZZ, 0DB38ZX, 0DB38ZZ, 0DB48ZX, 0DB48ZZ, 0DB58ZX, 0DB58ZZ, 0DB68ZX, 0DB68ZZ, 0DB78ZX, 0DB78ZZ, 0DB88ZX, 0DB88ZZ, 0DB98ZX, 0DB98ZZ, 0DBA8ZX, 0DBA8ZZ, 0DBB8ZX, 0DBB8ZZ, 0DBC8ZX, 0DBC8ZZ, 0DBE8ZX, 0DBE8ZZ, 0DBF8ZX, 0DBF8ZZ, 0DBG8ZX, 0DBG8ZZ, 0DBH8ZX, 0DBH8ZZ, 0DBJ8ZX, 0DBJ8ZZ, 0DBK8ZX, 0DBK8ZZ, 0DBL8ZX, 0DBL8ZZ, 0DBM8ZX, 0DBM8ZZ, 0DBN8ZX, 0DBN8ZZ
	CPT	43231, 43232, 43233, 43235, 43236, 43237, 43238, 43239, 43240, 43241, 43242, 43243, 43244, 43245, 43246, 43247, 43248, 43249, 43235, 43236, 43237, 43238, 43239, 43250, 43251, 43252, 45378, 45379,

© 2024 Michelson KA et al. *JAMA Network Open.*

		45380, 45381, 45382, 45384, 45385, 45386, 45388, 45389, 45390, 45391, 45392, 45393, 45395, 45397, 45398
Fontan surgery	ICD-10-PCS	061007P, 061007Q, 061007R, 061009P, 061009Q, 061009R, 06100AP, 06100AQ, 06100AR, 06100JP, 06100JQ, 06100JR, 06100KP, 06100KQ, 06100KR, 06100ZP, 06100ZQ, 06100ZR
Gastrostomy	CCS	71
Inflammatory bowel disease hospitalization	ICD-10-CM	K50*, K51*
Simple laceration repair	CPT	12001, 12002, 12004, 12005, 12006, 12007, 12011, 12013, 12014, 12015, 12016, 12017, 12018, 12020, 12021
	ICD-10-PCS	0JQ00ZZ, 0JQ03ZZ, 0JQ10ZZ, 0JQ13ZZ, 0JQ40ZZ, 0JQ43ZZ, 0JQ50ZZ, 0JQ53ZZ, 0JQ60ZZ, 0JQ63ZZ, 0JQ70ZZ, 0JQ73ZZ, 0JQ80ZZ, 0JQ83ZZ, 0JQ90ZZ, 0JQ93ZZ, 0JQB0ZZ, 0JQB3ZZ, 0JQC0ZZ, 0JQC3ZZ, 0JQD0ZZ, 0JQD3ZZ, 0JQF0ZZ, 0JQF3ZZ, 0JQG0ZZ, 0JQG3ZZ, 0JQH0ZZ, 0JQH3ZZ, 0JQJ0ZZ, 0JQJ3ZZ, 0JQK0ZZ, 0JQK3ZZ, 0JQL0ZZ, 0JQL3ZZ, 0JQM0ZZ, 0JQM3ZZ, 0JQN0ZZ, 0JQN3ZZ, 0JQP0ZZ, 0JQP3ZZ, 0JQQ0ZZ, 0JQQ3ZZ, 0JQR0ZZ, 0JQR3ZZ
Lumbar puncture	ICD-10-PCS<br>CPT	009U3ZX, 009U3ZZ<br>62270
Mechanical ventilation	ICD-10-PCS<br>CPT	5A1935Z, 5A1945Z, 5A1955Z<br>94002, 94003, 94004, 94005
Pneumonia hospitalization	CCS refined	J13*, J14*, J15*, J16*, J17*, J18*
Urea cycle defect hospitalization	ICD-10-CM	E722*
Closed reduction of radius	ICD-10-PCS	0PSHXZZ, 0PSJXZZ
	CPT	25560, 25565, 25574, 25575, 25600, 25605, 25606, 25607, 25608, 25609
Sickle cell anemia hospitalization	ICD-10-CM	D57*
Organ transplant	CCS	105, 176
Cerebral ventricular shunt	ICD-10-PCS	009600Z, 009630Z, 009640Z
Ventricular septal defect surgery	ICD-10-PCS	02QM*

* Chosen to represent complex congenital heart disease: hypoplastic left heart, pulmonary atresia, and tetralogy of Fallot only

© 2024 Michelson KA et al. *JAMA Network Open.*

eFigure 1. Utilization of Pediatric Resources by Hospitals in Each Pediatric Level

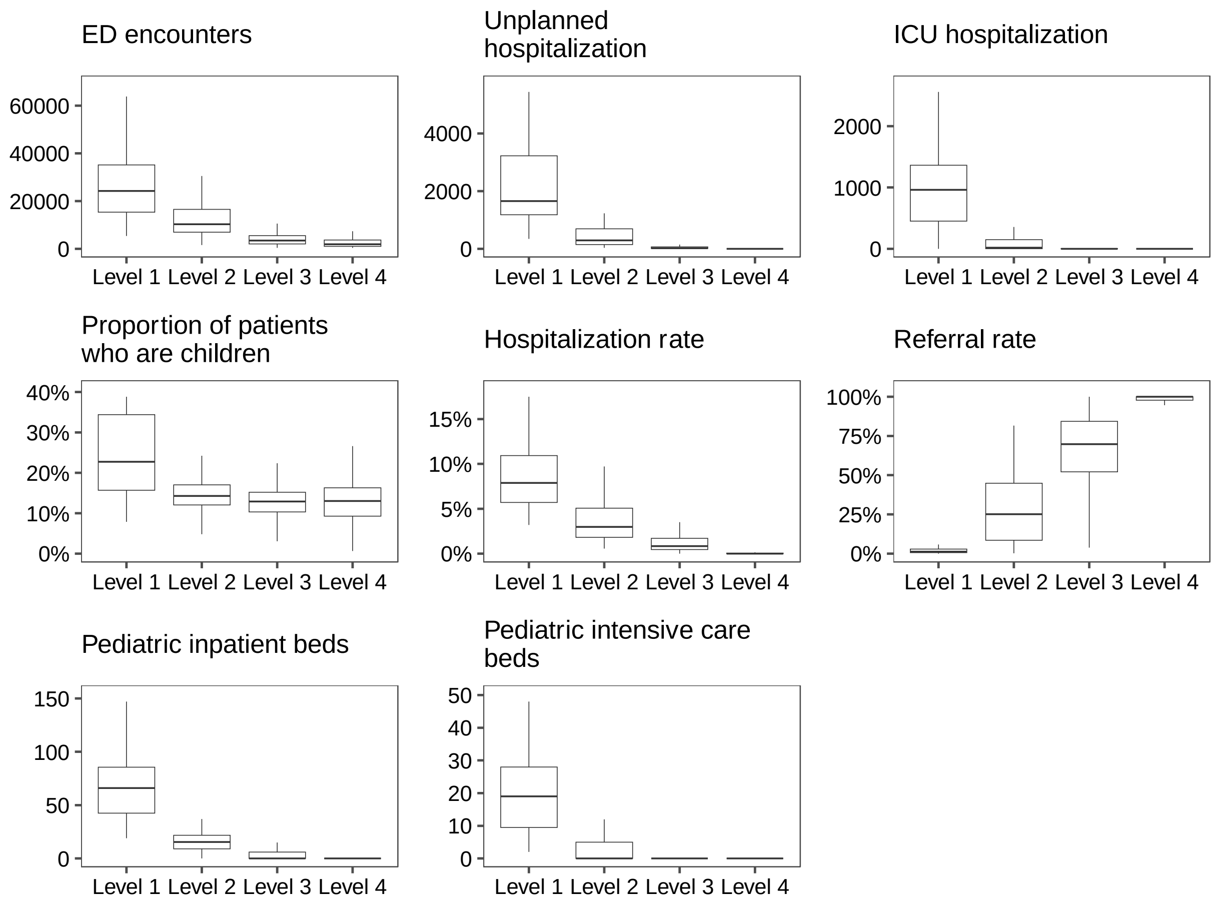

© 2024 Michelson KA et al. *JAMA Network Open.*

eFigure 2. Classification of Hospitals Into Functional Levels in the Main Analysis and in Two Sensitivity Analyses

Percentages of the hospitals in each level are shown in the heat map (blue: ≥75%, green: 50 to <75%, yellow: 25 to <50%, orange: >0 to <25%, white: 0%).

Main analysis

	Level 1 n=48 (5%)	Level 2 n=116 (11%)	Level 3 n=308 (29%)	Level 4 n=589 (56%)
CT scan	100	100	100	99
Simple laceration repair	100	95	93	92
Closed reduction of radius	98	97	73	46
Unplanned hospitalization	100	100	97	13
Appendectomy	100	98	69	13
Asthma hospitalization	100	100	78	<1
Pneumonia hospitalization	100	100	72	
Complex hospitalization	100	100	61	1
Lumbar puncture	100	99	45	12
Mechanical ventilation	100	84	16	3
ICU hospitalization	96	73	21	1
Sickle cell anemia hospitalization	98	63	6	
Gastrostomy	100	52	9	3
Diabetic ketoacidosis hospitalization	100	57	3	
Endoscopy	100	53	4	1
Inflammatory bowel disease hospitalization	100	22		
Chemotherapy hospitalization	98	11		
Congenital heart disease hospitalization	96			
Cystic fibrosis hospitalization	88	4		
Cerebral ventricular shunt	90	2		
Dialysis	77			
ECMO	71			
Organ transplant	52			
Ventricular septal defect surgery	48			
Urea cycle defect hospitalization	46			
Fontan surgery	44			

Sensitivity analysis: Based on children <12y

	Level 1 n=49 (5%)	Level 2 n=113 (11%)	Level 3 n=272 (26%)	Level 4 n=627 (59%)
CT scan	100	100	100	98
Simple laceration repair	100	95	93	92
Closed reduction of radius	96	95	67	37
Unplanned hospitalization	100	100	99	7
Appendectomy	100	92	56	5
Asthma hospitalization	100	100	80	
Pneumonia hospitalization	100	100	79	<1
Complex hospitalization	100	100	52	3
Lumbar puncture	100	96	39	9
Mechanical ventilation	100	77	10	4
ICU hospitalization	96	64	15	2
Sickle cell anemia hospitalization	98	62	4	
Gastrostomy	100	29	3	3
Diabetic ketoacidosis hospitalization	98	48		
Endoscopy	100	42	2	<1
Inflammatory bowel disease hospitalization	88	9		
Chemotherapy hospitalization	98	10		
Congenital heart disease hospitalization	92			
Cystic fibrosis hospitalization	76	4		
Cerebral ventricular shunt	84			
Dialysis	73			
ECMO	69			
Organ transplant	45			
Ventricular septal defect surgery	47			
Urea cycle defect hospitalization	39			
Fontan surgery	43			

Sensitivity analysis: 10+ procedures/yr defines capability

	Level 1 n=59 (6%)	Level 2 n=125 (12%)	Level 3 n=150 (14%)	Level 4 n=727 (69%)
CT scan	100	100	100	99
Simple laceration repair	100	94	91	91
Closed reduction of radius	95	86	45	18
Unplanned hospitalization	100	99	100	10
Appendectomy	98	87	60	2
Asthma hospitalization	100	97	67	<1
Pneumonia hospitalization	100	95	45	<1
Complex hospitalization	100	100	29	1
Lumbar puncture	100	58	1	1
Mechanical ventilation	100	37		1
ICU hospitalization	97	42	8	2
Sickle cell anemia hospitalization	95	32		
Gastrostomy	93	10	2	2
Diabetic ketoacidosis hospitalization	98	18		
Endoscopy	97	10		
Inflammatory bowel disease hospitalization	75			
Chemotherapy hospitalization	93			
Congenital heart disease hospitalization	56			
Cystic fibrosis hospitalization	54			
Cerebral ventricular shunt	49			
Dialysis	34			
ECMO	41			
Organ transplant	25			
Ventricular septal defect surgery	8			
Urea cycle defect hospitalization	7			
Fontan surgery	12			

Proportion of hospitals: 25% 50% 75%

© 2024 Michelson KA et al. *JAMA Network Open.*
