# Supplementary material for: Defining Levels of US Hospitals’ Pediatric Capabilities
Source: JAMA Netw Open. 2024 Jul 15;7(7):e2422196. doi: 10.1001/jamanetworkopen.2024.22196 (PMC11250363; doi:10.1001/jamanetworkopen.2024.22196)
Supplement: Supplement 2. — Data Sharing Statement [file jamanetwopen-e2422196-s002.pdf]

Data Sharing Statement

Michelson. Defining Levels of US Hospitals' Pediatric Capabilities. *JAMA Netw Open*. Published July 15, 2024. doi:10.1001/jamanetworkopen.2024.22196

Data
Data available: No

Additional Information
Explanation for why data not available: The data are available for purchase from the Healthcare Cost and Utilization Project
